# Synergistic Effects of Al Content and Cold Rolling on the Microstructural Evolution and Annealing Behavior of FeCoCrNi-Based High-Entropy Alloys

**DOI:** 10.3390/ma19214565

**Published:** 2026-10-29

**Authors:** Jiuming Pei, Danya Zhang, Chenyu Li, Zhisheng Nong

**Affiliations:** 1 School of Materials Science and Engineering, Shenyang Aerospace University, Shenyang 110136, China; 2 Key Laboratory of Fundamental Science for National Defense of Aeronautical Digital Manufacturing Process, Shenyang Aerospace University, Shenyang 110136, China

**Keywords:** high-entropy alloys, cold rolling, microstructures, annealing hardening

## Abstract

**Highlights:**

- Higher Al content shifts deformation from fragmentation to continuous banding.
- Rolling relieves segregation by fragmentation, not diffusion, hardening Al8 by 82%.
- Recovery softening and solute hardening set Al8/Al10 hardness at 328.3 and 364.4 HV.

**Abstract:**

This study investigates the effect of cold rolling and subsequent annealing on the microstructural evolution and hardness of Fe_16_Co_28_Cr_40-x_Ni_16_Al_x_ (x = 8, 10) high-entropy alloys (HEAs). The initial state alloys were subjected to 90% cold rolling followed by annealing at 800 °C for 15 and 30 min. Results indicate that both initial state alloys exhibit a typical face-centered cubic (FCC) dendritic morphology, with the higher Al content in the Al10 alloy promoting greater elemental segregation and yielding a higher initial hardness of 213.1 HV compared to 192.9 HV for the Al8 alloy. During 90% cold rolling, the dendrites of the Al8 alloy undergo severe fragmentation and refinement, reducing the dendritic feature size to 2.0 μm and leading to an 82% surge in hardness to 351.6 HV. Conversely, the Al10 alloy maintains continuously elongated dendritic bands due to higher deformation resistance, reaching a hardness of 334.0 HV. During subsequent annealing at 800 °C, the microstructural evolution pathways diverge. The Al8 alloy predominantly experiences recovery softening accompanied by structural dispersion, whereas the Al10 alloy exhibits continuous hardening due to substantial solid solution strengthening and high microstructural stability. These findings demonstrate that altering the Al content fundamentally shifts the initial deformation mode. Cold rolling plays a critical role in fragmenting the initial dendritic structure and in redistributing the solute-rich segregation domains to a finer scale, while driving substantial work hardening. Subsequent annealing governs the critical balance between recovery softening and structurally driven continuous hardening, highlighting the synergy of composition and processing in tuning the stability and performance of HEAs.

## 1. Introduction

High-entropy alloys (HEAs) are complex alloys composed of four or more major elements [1,2,3]. They fundamentally change traditional alloy design strategies and have attracted extensive attention from scholars [4,5,6]. Among these, the equiatomic FeCrMnNiCo system and its derivatives represent a cornerstone of HEA research. As revealed by Cantor et al. [7], these systems typically solidify into a dendritic structure predominantly consisting of a face-centered cubic (FCC) solid solution, which is a prevalent microstructural feature in CoCrFeNi-based HEAs in their initial state [8,9,10]. Elements such as Cr and Al typically tend to enrich within the primary dendritic trunks, whereas elements like Fe and Co are preferentially distributed and rejected into the interdendritic areas. While these initial state FCC solid solutions offer excellent uniform structural connectivity and stable solid-liquid interface advancement, their applications are frequently limited by inherent segregation and a need to optimize the balance between strength and deformation capabilities [11,12,13].

To tailor the mechanical performance and microstructural evolution of CoCrFeNi-based HEAs, adjusting the chemical composition, specifically through the incorporation of elements with large atomic radius such as Al, is a highly effective and widely employed metallurgical strategy [14,15,16]. The addition of Al dramatically alters the fundamental kinetics of solute redistribution during the final stages of solidification. Because of its large atomic radius, Al introduces severe lattice distortion into the FCC matrix, thereby enhancing the solid solution strengthening effect [17,18]. Furthermore, elevated Al content significantly increases supercooling at the solidification front. This thermodynamic shift promotes the nucleation rate, restricts subsequent grain growth, and leads to a refinement of the initial cast grain size, alongside an increased propensity for second-phase precipitation [19,20]. Consequently, varying the Al concentration directly dictates the localized deformation resistance of the matrix, fundamentally altering how the alloy responds to applied stress.

Beyond compositional tuning, deformation processing, particularly severe cold rolling, serves as a crucial method for modifying the initial state microstructural morphology and mitigating initial compositional segregation. The application of a large strain, such as a 90% thickness reduction, subjects the coarse primary initial state dendrites to intense normal compression and longitudinal tension. This intense plastic deformation effectively breaks down the original compositional boundaries and reduces the characteristic size of the segregation domains, indicating that the apparent mitigation of solidification-induced segregation is a mechanical process of microstructural fragmentation and redistribution, rather than thermally activated long-range atomic diffusion [21,22]. However, the morphological evolution pathway during cold rolling is highly dependent on the initial solute content of alloy. Alloys with relatively lower deformation resistance are prone to thorough structural fragmentation and refinement under intense shear stress, heavily disrupting dendrite continuity [23,24]. In contrast, alloys with higher solute additions and enhanced solid solution strengthening exhibit elevated deformation resistance, causing their primary dendrites to preferentially elongate into continuous, highly directional streamlined microstructures without complete fragmentation. This structural transformation leads to a surge in the density of grain boundaries, dendrite interfaces, and deformation bands, collectively driving substantial work hardening [25,26].

Following severe plastic deformation, annealing treatments are employed to trigger critical metallurgical processes such as recovery, structural rearrangement, and elemental redistribution. In heavily deformed HEAs, the high-density defect networks and massive stored strain energy provide a powerful driving force for these structural evolutions. During short-term annealing, the hardness and microstructural changes of the alloys are not dictated by a single factor, but rather by an intricate competition between multiple mechanisms, such as recovery softening, structural refinement, continuous solid solution strengthening, and the potential pinning effects of fine precipitated particles [27,28,29]. When recovery softening dominates, the alloy experiences a notable degradation in hardness. However, if the alloy possesses severe initial lattice distortion and high microstructural stability, thermal activation can drive localized solute atom redistribution that prevents softening and instead promotes a continuous hardening effect, allowing the alloy to retain its cold-rolled strength.

Despite the known individual influences of Al addition and cold working, the synergistic mechanisms through which varying Al contents dictate the specific deformation fragmentation pathways and subsequent short-term annealing behaviors in FeCoCrNi-based HEAs remain insufficiently understood. To address this gap, this study systematically investigates the Fe_16_Co_28_Cr_40-x_Ni_16_Al_x_ (x = 8, 10) HEAs. By subjecting the Al8 and Al10 alloys to 90% severe cold rolling followed by 800 °C annealing for 15 and 30 min, this research aims to elucidate how varying Al concentrations shift the deformation mode from thorough fragmentation to continuous banding. Furthermore, by tracking the microstructural and elemental evolution alongside hardness changes, this work aims to comprehensively reveal the underlying mechanisms of the critical competition between recovery softening and annealing hardening in these complex alloy systems.

## 2. Materials and Methods

The Fe_16_Co_28_Cr_40-x_Ni_16_Al_x_ (x = 8, 10) HEA was prepared by vacuum induction melting using high-purity elemental raw materials (≥99.9 wt%). Melting was conducted under an argon atmosphere, and the ingot was remelted at least five times to ensure chemical homogeneity. The cast ingots were homogenized at 1200 °C for 24 h followed by air quenching to obtain Al8 (x = 8) and Al10 alloys (x = 10). This condition is referred to as the initial state. The alloys were deformed at room temperature by multi-pass cold rolling to a 90% thickness reduction with a reduction of 0.3–0.5 mm per pass (final thickness~0.5 mm), and annealed at 1100 °C for 10 min. Subsequently, the Al8 and Al10 alloys before and after the cold rolling process were annealed at 800 °C for 15 or 30 min, and then cooled to room temperature in air. Microstructural characterization was performed on mechanically polished and etched samples.

Based on the calculation methods and our element composition, several characteristic parameters of the Al8 and Al10 alloys as shown in Table 1 [30]. The mixing entropy (∆S_mix_) are 1.51R and 1.53R (R is the gas constant, 8.314 J/(K·mol)). The mixing enthalpy (∆H_mix_) are −7.87 KJ/mol and −9.38 KJ/mol, and the atomic radii difference (δ) are 3.97% and 4.38%, the valence electron concentration (VEC) are 7.56 and 7.50. Therefore, this alloy can be classified as HEA. Surface preparation involved grinding with sandpapers followed by mechanical polishing and aqua regia etching. Microstructures and crystallographic features were characterized using an OLYMPUS BX51M optical microscope (OM) and Gemini SEM 300 field-emission scanning electron microscope (SEM) equipped with Super-X energy-dispersive X-ray spectroscopy (EDS) detectors. Phase identification was performed by ultima IV X-ray diffractometer (XRD) using Cu Kα radiation scanning from 20–90° in 2θ with a scanning rate of 5°/min.

The hardness of the alloys was evaluated by Vickers method under a load of 1 Kgf and a dwell time of 15 s on the rolling direction plane. Error bars in the results represent the standard deviation of at least three independent measurements made at randomly selected positions on two independently prepared specimens.

## 3. Results and Discussion

### 3.1. Microstructure of Alloys Before and After Cold Rolling

**Figure 1:**
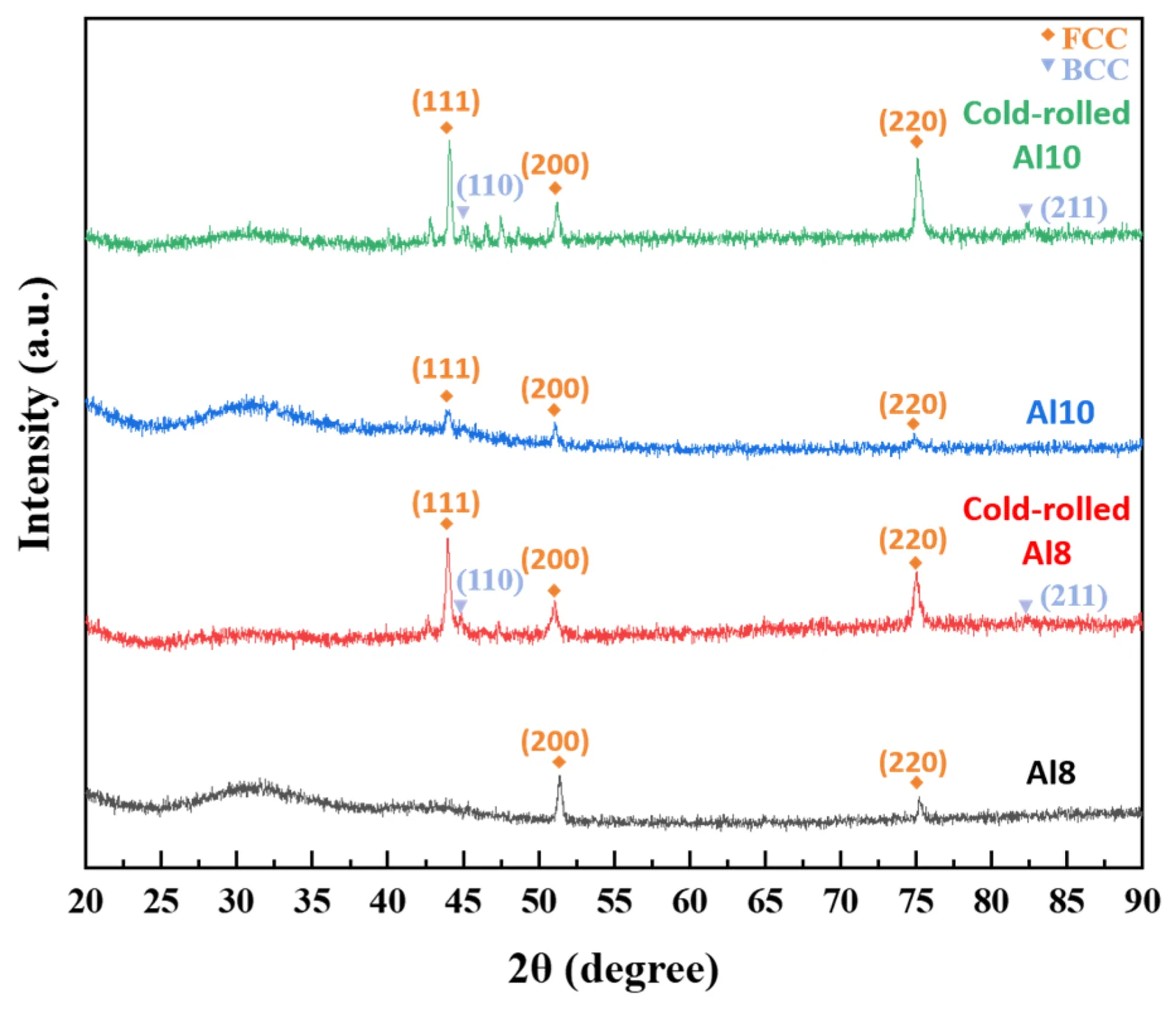
XRD patterns of the Al8 and Al10 alloys in the initial state and cold-rolled conditions.

Figure 1 shows the XRD patterns of the initial state and cold-rolled Al8 and Al10 alloys. It can be observed that, regardless of the Al content, the alloys in initial states exhibit a single primary FCC phase, with major diffraction peaks corresponding to the (111), (200), and (220) planes, indicating that in the initial state, the matrix is predominantly composed of a homogeneous FCC solid solution without distinct phase separation. After 90% cold rolling, the relative peak intensities of various crystal planes change. Diffraction peaks (110) and (211) of the body-centered cubic type (BCC-type) phase begin to emerge in the Al8 and Al10 samples.

**Figure 2:**
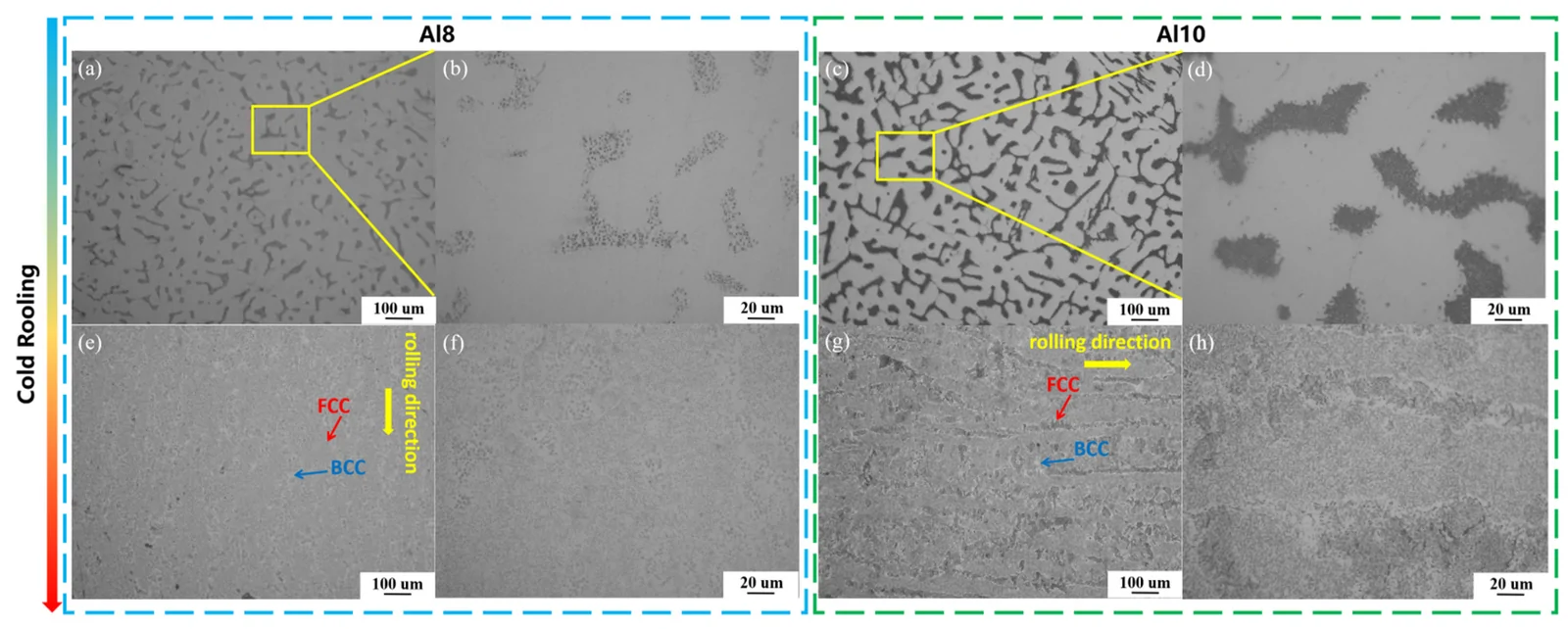
Microstructures of Fe_16_Co_28_Cr_40-x_Ni_16_Al_x_ HEA. (**a**) Al8 100×; (**b**) Al8 500×; (**c**) Al10 100×; (**d**) Al10 500×; (**e**) Al8 after cold rolling 100×; (**f**) Al8 after cold rolling 500×; (**g**) Al10 after cold rolling 100×; (**h**) Al10 after cold rolling 500×.

The Al8 alloy exhibits a typical initial state microstructural characterization. The alloy displays a distinct dendritic solidification morphology, composed of primary dendrite trunks and interdendritic regions, with localized development of secondary dendrite arm spacing. Cantor et al. [7] revealed that the equiatomic FeCrMnNiCo system forms a dendritic structure predominantly consisting of an FCC solid solution, indicating that dendritic morphology is a prevalent feature in the initial state of CoCrFeNi-based HEAs. As shown in Figure 2a, the dendrites exhibit an irregular network distribution with well-defined dendrite boundaries, suggesting preferential growth behavior during solidification. Owing to the relatively high cooling rate, the overall dendritic spacing is refined, resulting in a compact microstructure without obvious casting defects such as shrinkage cavities or microcracks. As shown in Figure 2b, the dendrite interiors are homogeneous, whereas a small quantity of bright white granular second-phase particles is dispersed in the interdendritic regions, indicating minor elemental segregation during the final stages of solidification. Dendritic feature size analysis shows that the dendritic feature size of the initial state Al8 alloy is 29.6 μm, concentrated mainly in the range of 20–40 μm, which confirms good microstructural homogeneity. Overall, the initial state Al8 alloy demonstrates characteristic features of dendritic FCC HEAs with high structural uniformity, providing a favorable initial microstructural state for subsequent cold rolling and annealing recrystallization investigations.

As shown in Figure 2c, the initial state microstructure of the Al10 alloy also exhibits a typical dendritic morphology. Compared to the Al8 alloy, the dendritic features in the Al10 alloy are more pronounced, characterized by significantly coarsened primary dendrite trunks and more distinct interdendritic regions. This indicates that the increased Al content enhances solute redistribution during solidification, thereby exacerbating elemental dendritic segregation. As shown in Figure 2d, a higher density of fine granular or island-like particles is dispersed within the interdendritic regions, locally tending towards a continuous distribution. The dendritic feature size of the initial state Al10 alloy is 28.9 μm, predominantly distributed in the range of 20–40 μm. Compared with the Al8 alloy, the Al10 alloy displays a broader dendritic feature size distribution accompanied by a higher fraction of fine dendrites. This minor refinement may be attributed to the elevated Al content, which increases supercooling, promotes the nucleation rate, and restricts subsequent dendrite growth [31]. In addition, localized dendritic interlacing is observed, which may reflecte strong competitive growth among dendrites with different crystallographic orientations during solidification.

After 90% cold rolling, the original coarse initial state dendrites of both Al8 and Al10 alloys undergo severe plastic deformation, becoming strongly elongated and refined along the rolling direction. As shown in Figure 2e,f, the original dendritic contours of the Al8 alloy almost completely vanish, exhibiting a dense deformation microstructure. This indicates that its primary dendrite trunks have experienced thorough refinement during the rolling process, with the dendritic feature size reduced to 2.0 μm. In contrast, the Al10 alloy in Figure 2g,h displays a highly directional streamlined microstructure extending along the rolling direction. Although drastically stretched, the original primary dendrite trunks maintain good continuity, with the dendritic feature size decreasing to 4.3 μm. This disparity in microstructural evolution is primarily attributed to the varying Al contents. The strong solid solution strengthening effect induced by the Al addition significantly enhances the deformation resistance of the Al10 matrix, causing its dendrites to preferentially elongate integrally along the rolling direction [32]. Conversely, the relatively lower deformation resistance of the Al8 alloy makes it more prone to fragmentation under intense shear stress.

**Figure 3:**
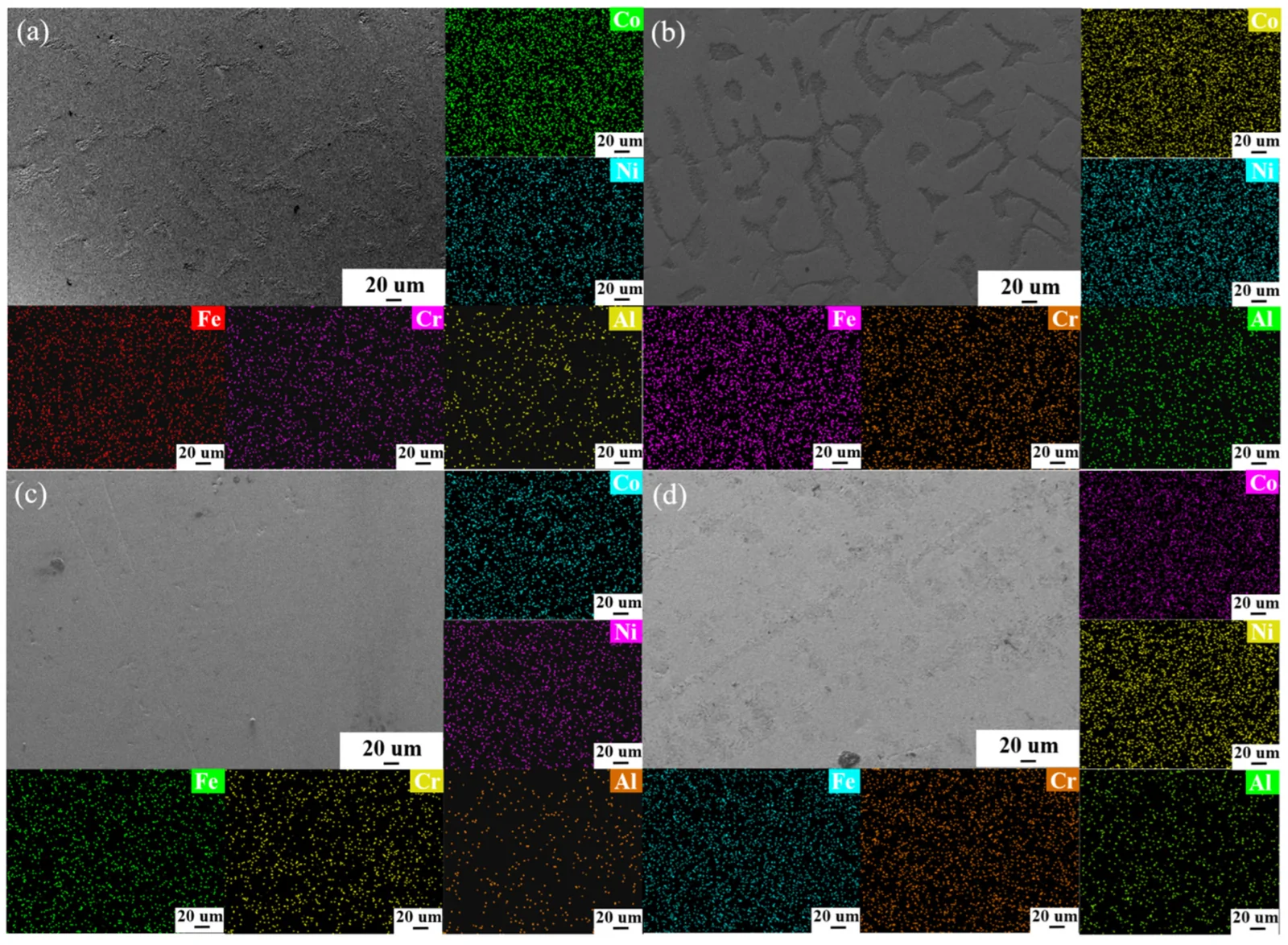
SEM micrographs and corresponding EDS elemental maps of Fe_16_Co_28_Cr_40-x_Ni_16_Al_x_ HEA. (**a**) Al8 alloy; (**b**) Al10 alloy; (**c**) Al8 alloy after cold rolling; (**d**) Al10 alloy after cold rolling.

As shown in Figure 3a, the initial state Al8 alloy exhibits mild segregation. Elements such as Cr and Al tend to enrich within the dendritic regions, whereas Fe and Co are preferentially distributed in the interdendritic areas. This phenomenon is predominantly governed by the differing solid-liquid partition coefficients of the constituent elements during the solidification of HEAs. Owing to the lower Al content in the Al8 alloy, solute redistribution remains limited, resulting in subtle compositional contrast between the dendrite trunks and interdendritic regions. Consequently, the matrix retains a homogeneous single-phase FCC solid solution structure without distinct phase separation. Localized compositional fluctuations can noticeably influence local phase stability and interfacial behavior, providing a favorable initial state for microstructure fragmentation and elemental redistribution during subsequent cold rolling. As shown in Figure 3b, the EDS mapping of the Al10 alloy demonstrates that increased Al addition accelerates localized elemental enrichment. The large atomic radius of Al introduces severe lattice distortion and alters the kinetics of solute redistribution during the final stages of solidification, thereby expanding the chemical gradient between dendritic and interdendritic zones. Overall, while the Al10 alloy maintains a predominantly FCC dendritic structure in its initial state, the higher Al content intensifies segregation, promotes microstructural refinement.

Cold rolling also modifies the spatial distribution of the solute-rich and solute-lean regions inherited from solidification, in addition to changing the microstructural morphology. As shown in Figure 3c,d, the distributions of Fe, Co, Cr, Ni, and Al in both alloys appear more continuous after cold rolling than in the initial state. As shown in Figure 3c, the dendrite trunks and interdendritic regions are fragmented to such an extent that the original compositional boundaries are no longer resolvable at the scale of the EDS maps. As shown in Figure 3d, the elemental distribution likewise becomes less contrasted, yet band-like compositional heterogeneities aligned with the rolling direction remain discernible, consistent with the retention of continuously elongated dendrite bands. Therefore, the decrease in the contrast of the distribution map indicates the fragmentation and mechanical redistribution of the region. Under this constraint, the grains of the Al8 alloy are finer and more evenly distributed, and the distribution pattern of its phases is more refined and dispersed than that of the Al10 alloy.

### 3.2. Effect of Annealing on Microstructures of Alloys Before and After Cold Rolling

**Figure 4:**
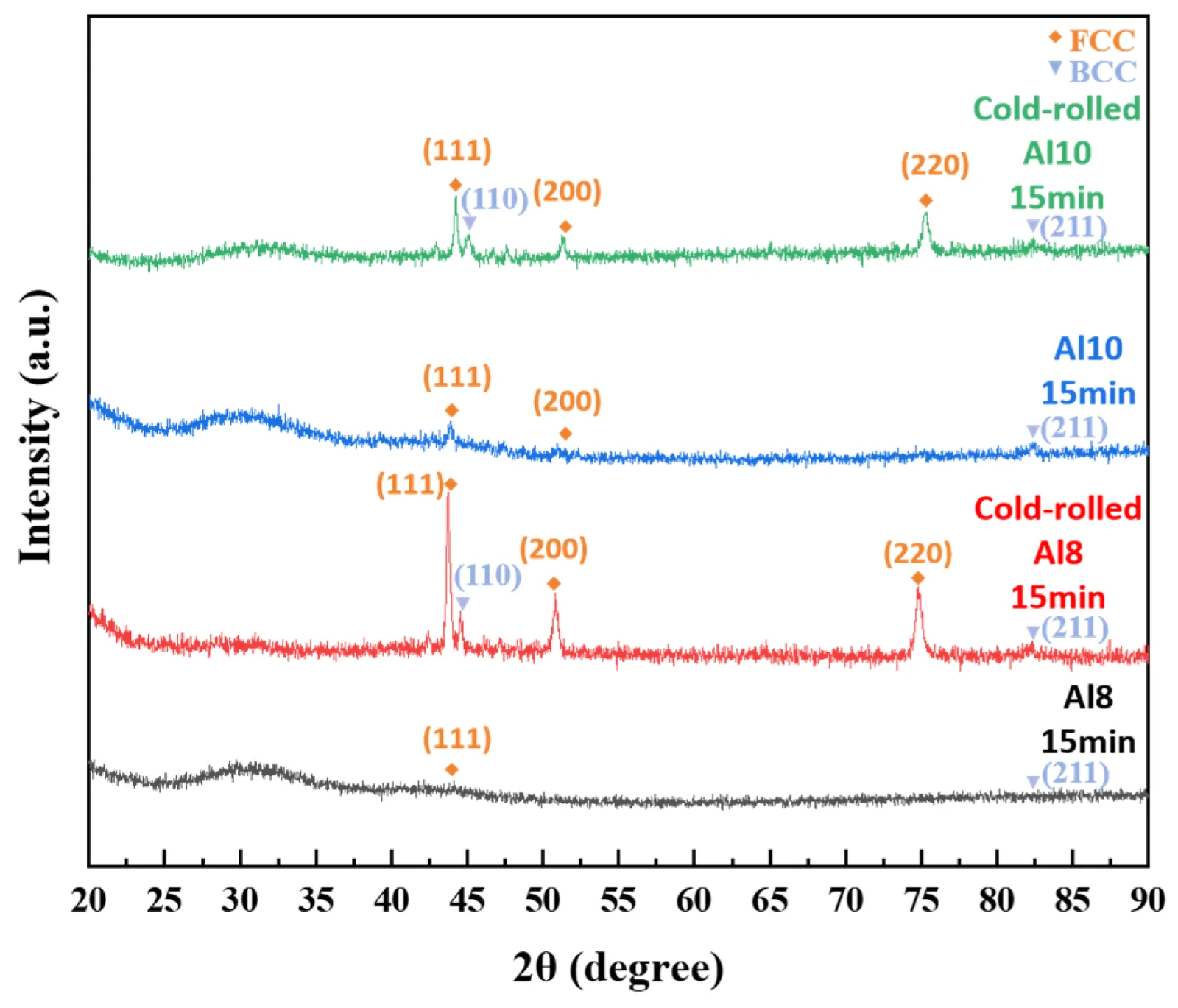
XRD Patterns of Fe_16_Co_28_Cr_40-x_Ni_16_Al_x_ HEA after 800 °C annealing for 15 min.

Figure 4 displays the XRD patterns of the alloys after annealing at 800 °C for 15 min. Diffraction peaks of the BCC-type phase emerge after annealing in initial states. Diffraction peaks are more distinct in the cold-rolled condition and in the Al10 alloy, a trend that appears to be associated with the higher Al content and with the stored deformation energy introduced by cold rolling.

**Figure 5:**
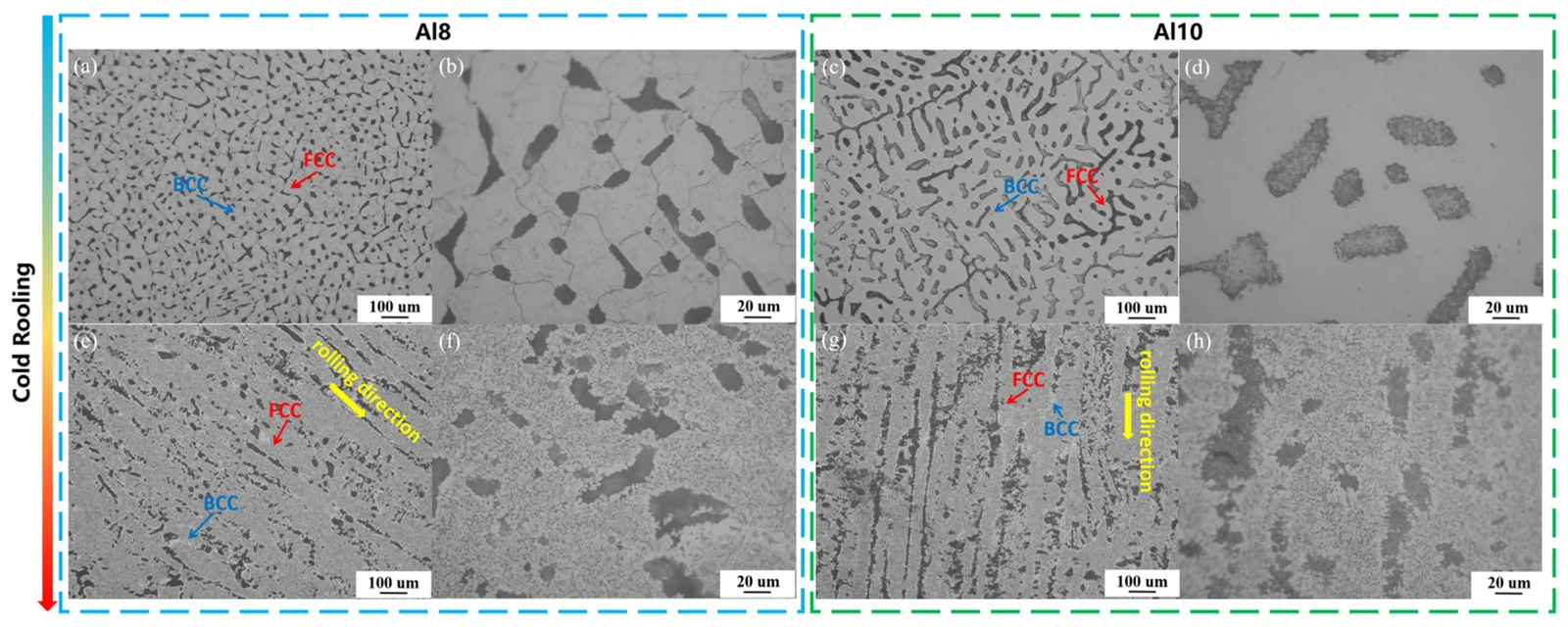
Microstructures of Fe_16_Co_28_Cr_40-x_Ni_16_Al_x_ HEA after 800 °C annealing for 15 min. (**a**) Al8 100×; (**b**) Al8 500×; (**c**) Al10 100×; (**d**) Al10 500×; (**e**) Al8 after cold rolling 100×; (**f**) Al8 after cold rolling 500×; (**g**) Al10 after cold rolling 100×; (**h**) Al10 after cold rolling 500×.

Microstructural observations of the undeformed Al8 and Al10 alloys annealed at 800 °C for 15 min reveal that both alloys retain distinct initial state dendritic features. Owing to the absence of prior plastic deformation, the stored energy may be relatively low, preventing significant recrystallization during the short 15 min annealing process. Consequently, microstructural evolution is dominated by solute element diffusion-assisted redistribution, localized stress relaxation, and subtle structural rearrangement, rather than recrystallization. For the Al8 alloy, the dendritic structure is uniformly distributed. As shown in Figure 5a, the interdendritic regions exhibit discontinuous granular, short rods, or irregular blocky geometries without noticeable directionality. As shown in Figure 5b, blunted dendrite boundaries and a weakened contrast between the dendritic cores and interdendritic regions are revealed, indicating that short-term annealing partially alleviates dendritic segregation. The dendritic feature size of the annealed Al8 alloy is 18.6 μm, predominantly concentrated within the 15–25 μm range, exhibiting a high degree of structural uniformity. As shown in Figure 5c, the Al10 alloy displays a coarser and more continuous dendritic morphology in contrast, characterized by networked, elongated, or island-like distributions. As shown in Figure 5d, the rough dendritic edges reflect persistent localized segregation. The dendritic feature size of the annealed Al10 alloy is 12.8 μm, mostly ranging from 5–10 μm, along with larger dendrites of 15–25 μm that result in a broader size distribution. This discrepancy is attributed to the higher Al content in Al10, which enhances solute redistribution during solidification and accentuates initial dendritic segregation. Given the limited annealing time of 15 min, solute diffusion-assisted redistribution fails to eliminate this segregation completely, thereby preserving the continuous dendritic morphology in the Al10 alloy.

After 90% cold rolling followed by annealing at 800 °C for 15 min, both Al8 and Al10 alloys lose their original equiaxed dendritic morphologies and present typical deformed microstructures aligned along the rolling direction, appearing as banded and fibrous structures. The elongated and flattened dendrites undergo recovery and rearrangement, however, the deformation features persist due to the short annealing time. In the Al8 alloy, dark interdendritic regions appear as interrupted bands along the rolling direction. As shown in Figure 5e, these regions are partially fragmented into fine particles and short rods. As shown in Figure 5f, the dendritic feature size of cold-rolled and annealed Al8 decreases dramatically to 4.5 μm, which is heavily concentrated in the 3–7 μm range, representing substantial microstructural refinement compared to its undeformed alloy. As shown in Figure 5g, the Al10 alloy similarly displays strong directional banding. As shown in Figure 5h, dark constituents are continuously distributed along the rolling direction, while localized regions show fine dispersion. The cold-rolled and annealed Al10 alloy achieves a dendritic feature size of 4.1 μm, tightly distributed within the 3–4 μm range.

Cold rolling fundamentally modifies the microstructural evolution path during subsequent annealing. In undeformed alloys, the minimal stored energy restricts the driving force to chemical concentration gradients and interfacial energy, resulting in slow structural relaxation without pronounced recrystallization. Conversely, 90% cold rolling introduces high density deformation bands and stored energy, which accelerate recovery and local microstructural rearrangement. This leads to extensive dendrite fragmentation, drastic microstructural refinement, and well-defined banded structures. Furthermore, Al content indirectly dictates the structural evolution pattern post cold rolling. The Al8 alloy primarily undergoes a fragmentation, refinement, and dispersion path, where dendrites are readily broken down into discrete particles and short bands. The increase in Al content exacerbates the solute segregation during the initial solidification, enhances lattice distortion and solute strengthening, and alters the phase composition and transformation kinetics of the alloy. These combined effects enhance the matrix deformation resistance of the Al10 alloy, leading to continuous elongation of the primary dendrite trunks during cold rolling and annealing and resulting in an elongation, banding, and localized refinement microstructural evolution pathway.

**Figure 6:**
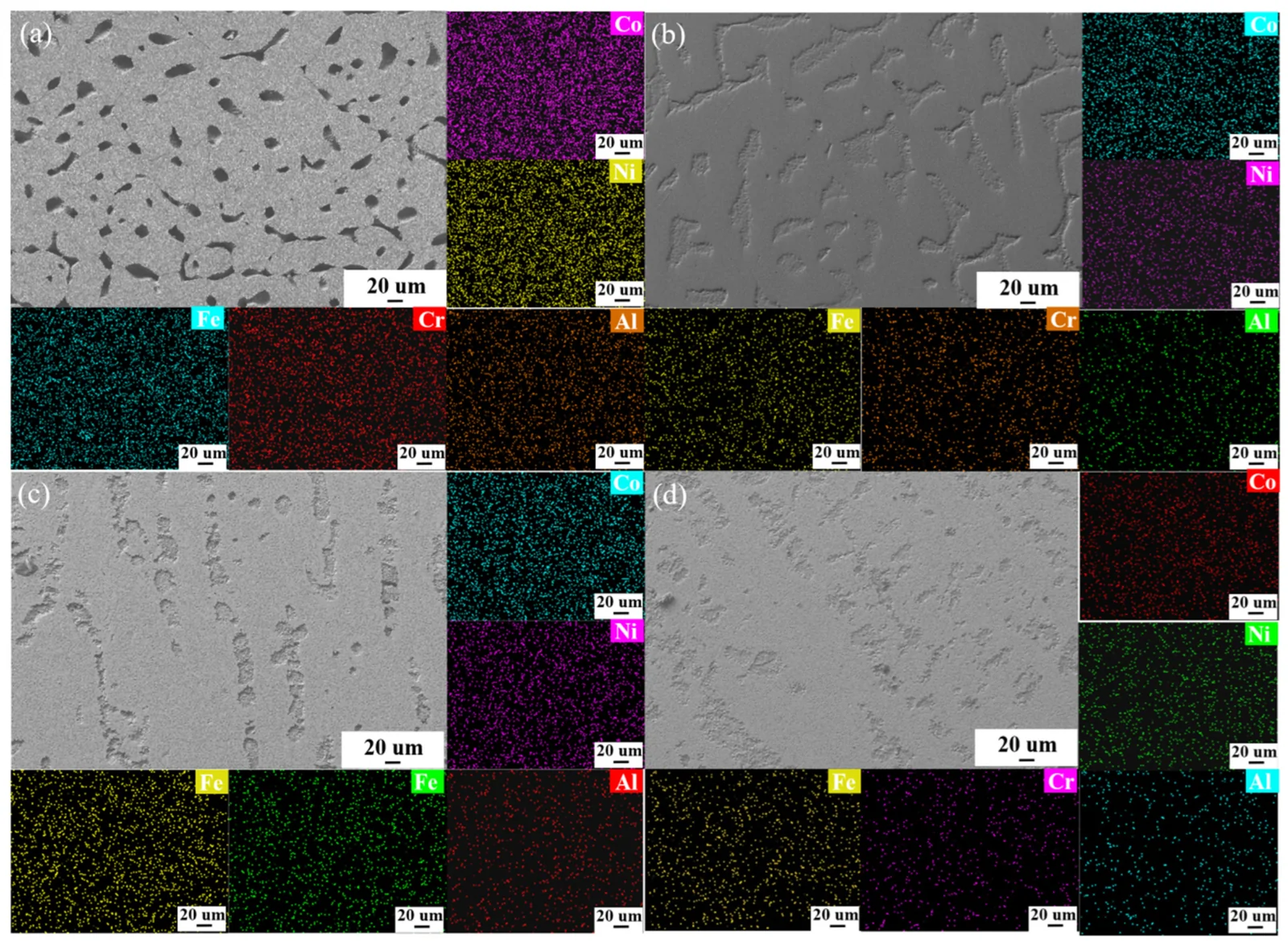
SEM micrographs and corresponding EDS elemental maps of Fe_16_Co_28_Cr_40-x_Ni_16_Al_x_ HEA after 800 °C annealing for 15 min. (**a**) Al8 alloy; (**b**) Al10 alloy; (**c**) Al8 alloy after cold rolling; (**d**) Al10 alloy after cold rolling.

To further elucidate the element distribution and segregation behavior during the annealing process, EDS analysis was conducted. As shown in Figure 6a, the undeformed Al8 alloy annealed for 15 min exhibits a generally uniform element distribution, with only slight elemental enrichment in the interdendritic regions. As shown in Figure 6b, the undeformed Al10 alloy under the same annealing conditions displays more pronounced elemental segregation, where solute elements are prominently enriched along the continuous dendrite trunks and networked interdendritic regions. This further confirms that a higher Al content exacerbates the initial dendritic segregation during solidification, which cannot be entirely eliminated by short-term annealing. Cold rolling significantly alters the spatial distribution morphology of the elements. As shown in Figure 6c, the EDS map of the cold-rolled and annealed Al8 alloy reveals that the enriched elements are dispersedly distributed following dendrite fragmentation, presenting fine granular and short-banded features consistent with the microstructure. This indicates that the deformation introduced by cold rolling redistributes the solute-rich domains into a finer and more dispersed arrangement. As shown in Figure 6d, the elemental distribution of the cold-rolled and annealed Al10 alloy exhibits strong rolling directionality, with solute elements continuously enriched in bands along the rolling direction. This is consistent with the continuous banded morphology described above.

**Figure 7:**
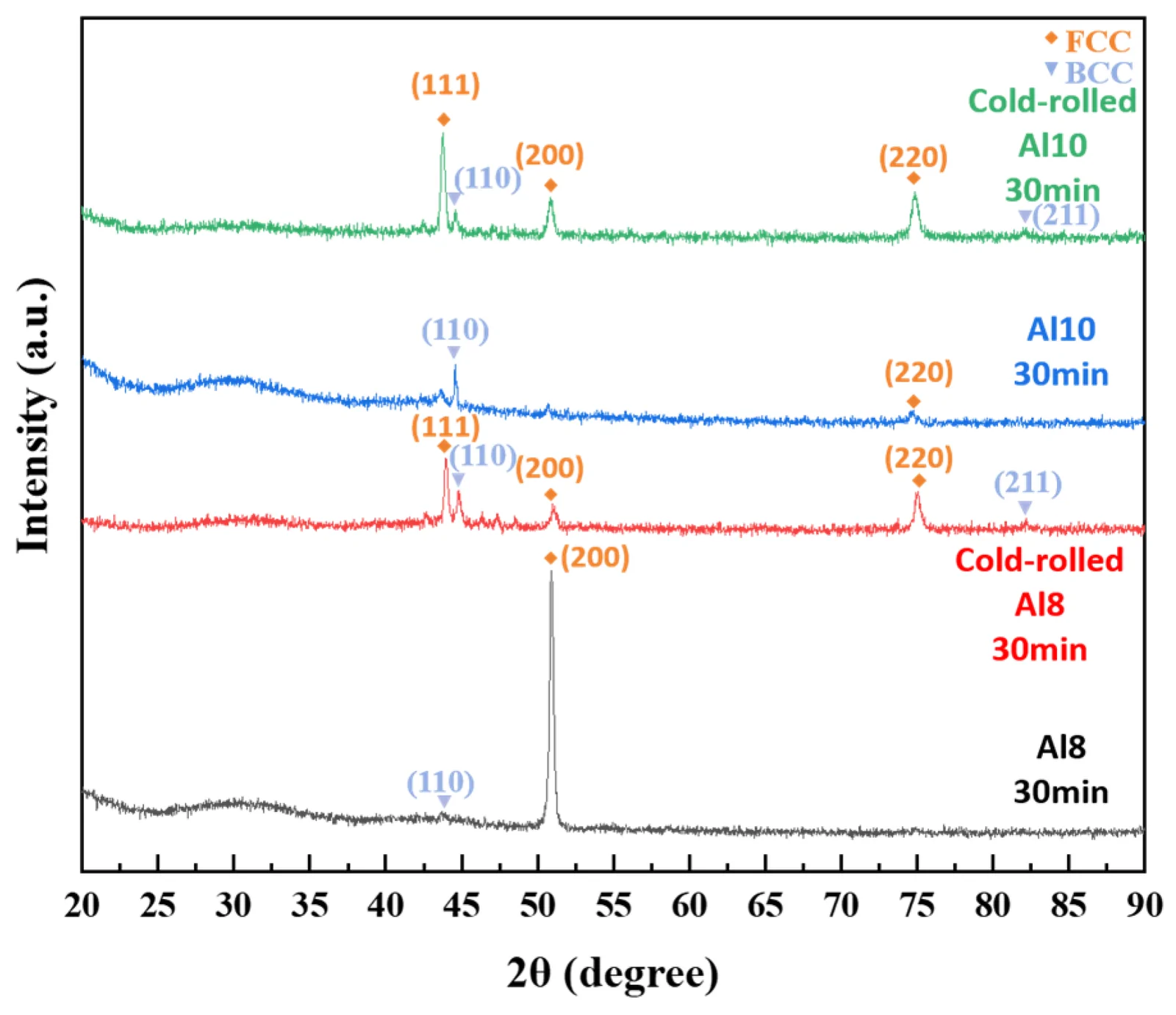
XRD Patterns of Fe_16_Co_28_Cr_40-x_Ni_16_Al_x_ HEA after 800 °C annealing for 30 min.

Figure 7 presents the XRD patterns after the annealing time extended to 30 min. With the increase in annealing time, the diffraction peaks of the BCC-type phase become more distinct. Additionally, the (200) FCC peak of the undeformed Al8 alloy is exceptionally sharp and highly intense, indicating the possible existence of a strong preferred annealing orientation. This (200) annealing texture is likely attributed to selective grain growth driven by the minimization of interfacial energy during the short-term annealing of the undeformed dendritic structure.

**Figure 8:**
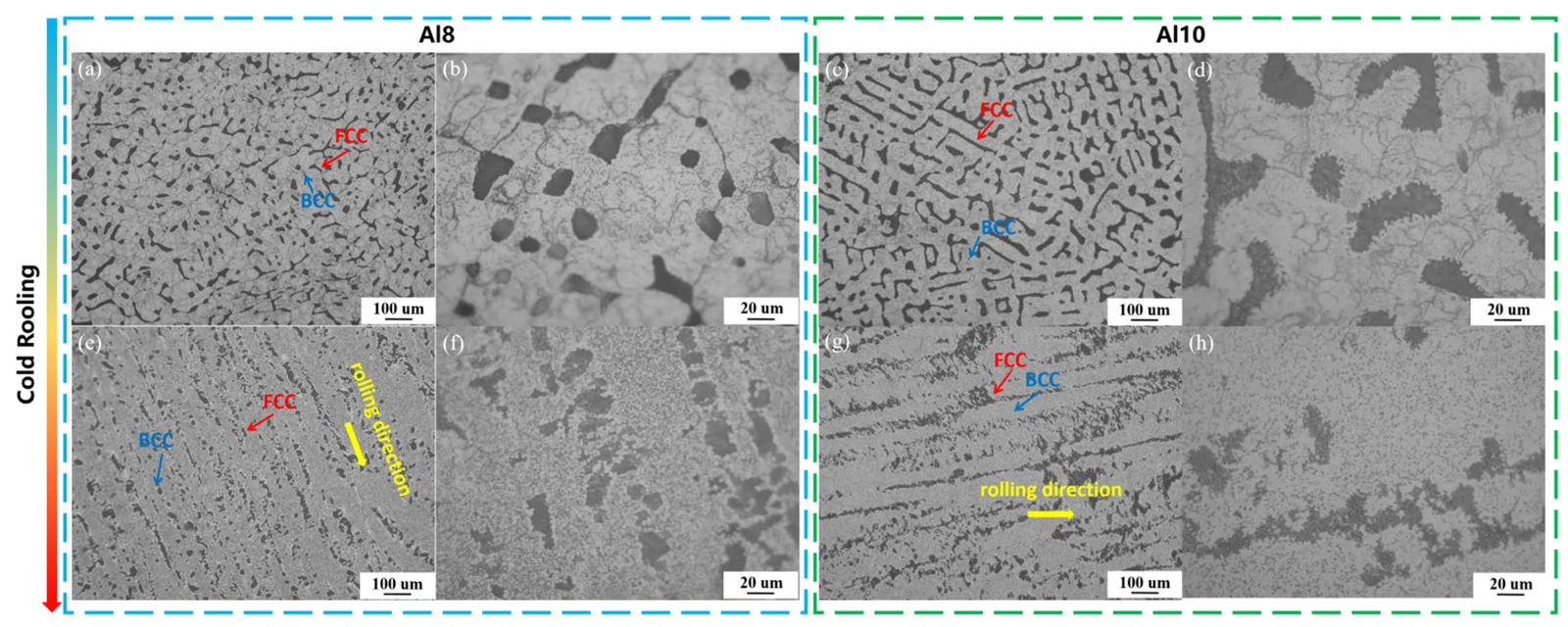
Microstructure of Fe_16_Co_28_Cr_40-x_Ni_16_Al_x_ HEA after 800 °C annealing for 30 min. (**a**) Al8 100×; (**b**) Al8 500×; (**c**) Al10 100×; (**d**) Al10 500×; (**e**) Al8 after cold rolling 100×; (**f**) Al8 after cold rolling 500×; (**g**) Al10 after cold rolling 100×; (**h**) Al10 after cold rolling 500×.

As shown in Figure 8a–d, the undeformed Al8 and Al10 alloys annealed at 800 °C for 30 min retain prominent initial state dendritic microstructures. Compared with the 15 min annealed state, extended holding time promotes elemental diffusion-assisted redistribution and interfacial migration, rendering dendrite boundaries more distinct. However, due to the lack of the stored energy provided by plastic deformation, recrystallization may still not be able to obtain sufficient driving force. As shown in Figure 8a,b, dark interdendritic regions in the Al8 alloy are dispersedly distributed as short rods, particles, and irregular blocks without preferred orientation. The dendrite boundaries undergo further blunting, and long interdendritic features segment into shorter, isolated morphologies, indicating enhanced solute redistribution and interfacial relaxation. The dendritic feature size of the Al8 alloy is 12.1 μm, predominantly within 10–14 μm. In contrast, as shown in Figure 8c,d, the Al10 alloy displays coarsened interdendritic regions and enhanced dendritic trunk continuity, forming localized networked and lamellar configurations. The rough interdendritic edges and expanded dark regions signify aggregation and coarsening driven by interfacial energy reduction. The dendritic feature size of the Al10 alloy reaches 10.7 μm (concentrated at 10–15 μm), indicating a distinct trend of dendrite coarsening. Theoretically, microstructural evolution in undeformed alloys is co-governed by elemental diffusion-assisted redistribution and interfacial energy minimization, prolonged annealing homogenizes compositional gradients between dendritic trunks and interdendritic regions while driving feature coarsening to reduce system energy. Comparatively, the Al8 alloy exhibits superior structural homogeneity, whereas the Al10 alloy retains stronger residual solidification segregation. This is attributed to the increased Al content, which exacerbates solute redistribution during solidification and enhances matrix stability via solid solution strengthening, thereby retarding the dissolution of original dendritic morphology.

As depicted in Figure 8e–h, the 90% cold rolled Al8 and Al10 alloys after annealing at 800 °C for 30 min maintain pronounced deformation texturing, characterized by fibrous and banded structures aligned along the rolling direction. Therefore, the evolution process may be mainly controlled by the processes of recovery, dendrite growth and local coarsening. As shown in Figure 8e,f, as the stored strain energy is released, the discontinuous zones and fine particles of the cold-rolled Al8 alloy become coarser and more rounded along the rolling direction. The dendritic feature size increases from 3–7 μm (15 min annealing) to 8.9 μm, mainly concentrated in the range of 5–12 μm. As shown in Figure 8g,h, for the cold-rolled Al10 alloy, the continuity of parallel bands is well preserved. Although its dendritic feature size expands from 3–4 μm (15 min annealing) to 8.1 μm, mainly concentrated in the range of 5–7 μm, the extent of dendrite growth remains lower than that in the Al8 alloy. This implies that the higher Al concentration and strong solid solution strengthening in Al10 enhance matrix stability and suppress dendrite growth.

A comprehensive comparison demonstrates that the microstructural evolution during annealing is synergistically governed by cold deformation, annealing time, and Al content. Primarily, heavy cold rolling may introduce high defect densities and deformation bands, which remarkably refine the annealed dendrite size, such as reducing the Al10 dendrite size from 10–15 μm in the undeformed state to 5–7 μm in the cold rolled state, while imparting strong morphological anisotropy. Furthermore, extending the annealing time from 15 to 30 min may shift the dominant mechanism from initial strain recovery to boundary migration and subgrain coalescence, manifested as boundary blunting and an increase in dendrite size. Additionally, the elevated Al content under this annealing process plays a pivotal role, it not only intensifies the initial solidification segregation but also enhances matrix stability against boundary migration via strong solid solution strengthening, thereby endowing the Al10 alloy with superior microstructural stability [33].

### 3.3. Effect of Annealing on the Hardness of Alloys Before and After Cold Rolling

**Figure 9:**
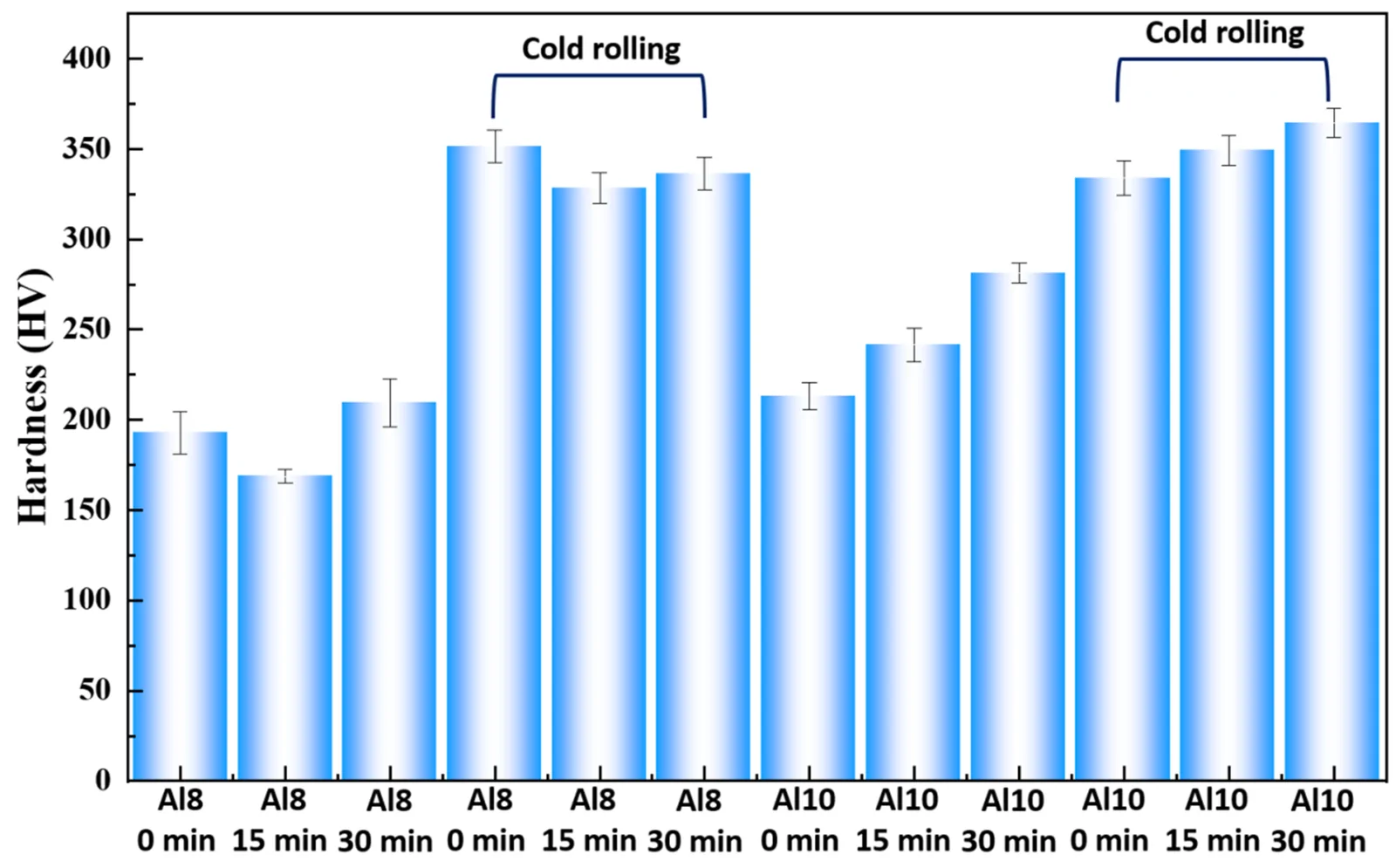
Hardness of the alloy after annealing treatment for both the initial state and the cold-rolled state, error bars represent the standard deviation of at least three independent measurements.

Figure 9 presents the hardness variations of the Al8 and Al10 alloys before and after 90% cold rolling. In the initial state, the hardness of the Al10 alloy is 213.1 HV, which is higher than the 192.9 HV of the Al8 alloy, mainly attributed to the severe lattice distortion and solid solution strengthening effect induced by the higher Al content. However, after 90% severe cold rolling, the coarse primary initial state dendrites are elongated, flattened, and subjected to intense fragmentation and refinement. This microstructural transformation might lead to a surge in the density of dendrite boundaries, dendrite interfaces, and deformation bands, substantially enhancing the resistance of the alloy to localized plastic deformation. Notably, the hardness of the cold-rolled Al8 alloy surges to 351.6 HV with an 82% increase, surpassing the 334.0 HV hardness and 57% increment of the Al10 alloy. This might be because the continuity of dendrite trunks in the Al8 alloy is more thoroughly disrupted during cold rolling, yielding a higher degree of dendrite arm fracture and a marked decrease in dendritic feature size, which would be expected to produce a stronger structural refinement strengthening and work hardening response. In contrast, the Al10 alloy retains more continuous dendrite trunks, leading to relatively weaker refinement. Therefore, the high initial hardness of the Al10 alloy relies on solid solution strengthening, whereas the Al8 alloy exhibits superior structural refinement and work hardening ability under large strains.

The high-density defects and work hardening effects introduced by the large strain are effectively retained during subsequent annealing at 800 °C. However, as shown in Figure 9, the annealing hardness evolution trajectories of the two alloys exhibit significant differences governed synergistically by composition and work hardening. The hardness variation during annealing is not dictated by a single factor but results from the combined effects of recovery softening, structural refinement, and solid solution strengthening. Under identical annealing durations, the cold-rolled alloys consistently exhibit substantially higher hardness values than the undeformed alloys. Specifically, for the Al8 alloy, the hardness of the undeformed alloy decreases to 168.8 HV after 15 min of annealing due to internal stress release, and then recovers to 209.4 HV after 30 min of annealing. For the cold-rolled Al8 alloy, the hardness drops from a peak of 351.6 HV to 328.3 HV with a 6.62% decline after 15 min of annealing, indicating that short-time heat treatment releases partial stored deformation energy and induces recovery softening. After 30 min of annealing, the hardness slightly rebounds to 336.5 HV, which was 4.29% lower than the state before annealing. For the Al10 alloy, the hardness of the undeformed Al10 alloy increases monotonically, from 213.1 HV in the initial state to 241.6 HV after 15 min and 281.2 HV after 30 min. Because these specimens contain little stored deformation energy. It is more plausibly associated with thermally activated, compositionally driven processes, namely the progressive formation of the BCC-type phase detected by XRD, the accompanying elemental redistribution, and the resulting increase in the lattice distortion of the FCC matrix. The cold-rolled Al10 alloy similarly displays a pronounced hardening trend, rising from 334.0 HV before annealing to 349.2 HV at 15 min with a 4.55% increase, and further surging to 364.4 HV at 30 min with an increment of 9.10%. The higher hardness indicate that the stored deformation energy and the refined, banded structure accelerate or add to this mechanism, rather than being its sole origin. In summary, for the Al8 alloy, short-term annealing might be dominated by recovery softening caused by dislocation annihilation and stored energy release. However, as the annealing time is extended to 30 min, residual deformation bands and refined structures that might not completely disappeared continue to impede dislocation slip. Combining with localized subgrain stabilization and the pinning effect of fine particles, prompts a moderate hardness recovery. In contrast, the extremely high Al content in the Al10 alloy triggers more severe lattice distortion, endowing it with significant solid solution strengthening. During annealing, thermal activation drives localized solute atom redistribution and microstructural adjustments in interdendritic regions. For instance, it may result in precipitation strengthening achieved through the precipitation of second phases [34,35]. Under the present short-term annealing conditions at 800 °C, this annealing hardening appears to outweigh the softening associated with the limited structural recovery, enabling the Al10 alloy to retain most of its cold-rolled strength and to exhibit continuous hardening. This behavior is attributed to the combined influence of the higher Al content, the associated solidification segregation and the constitutional features that this addition brings about, rather than to the Al concentration alone.

**Figure 10:**
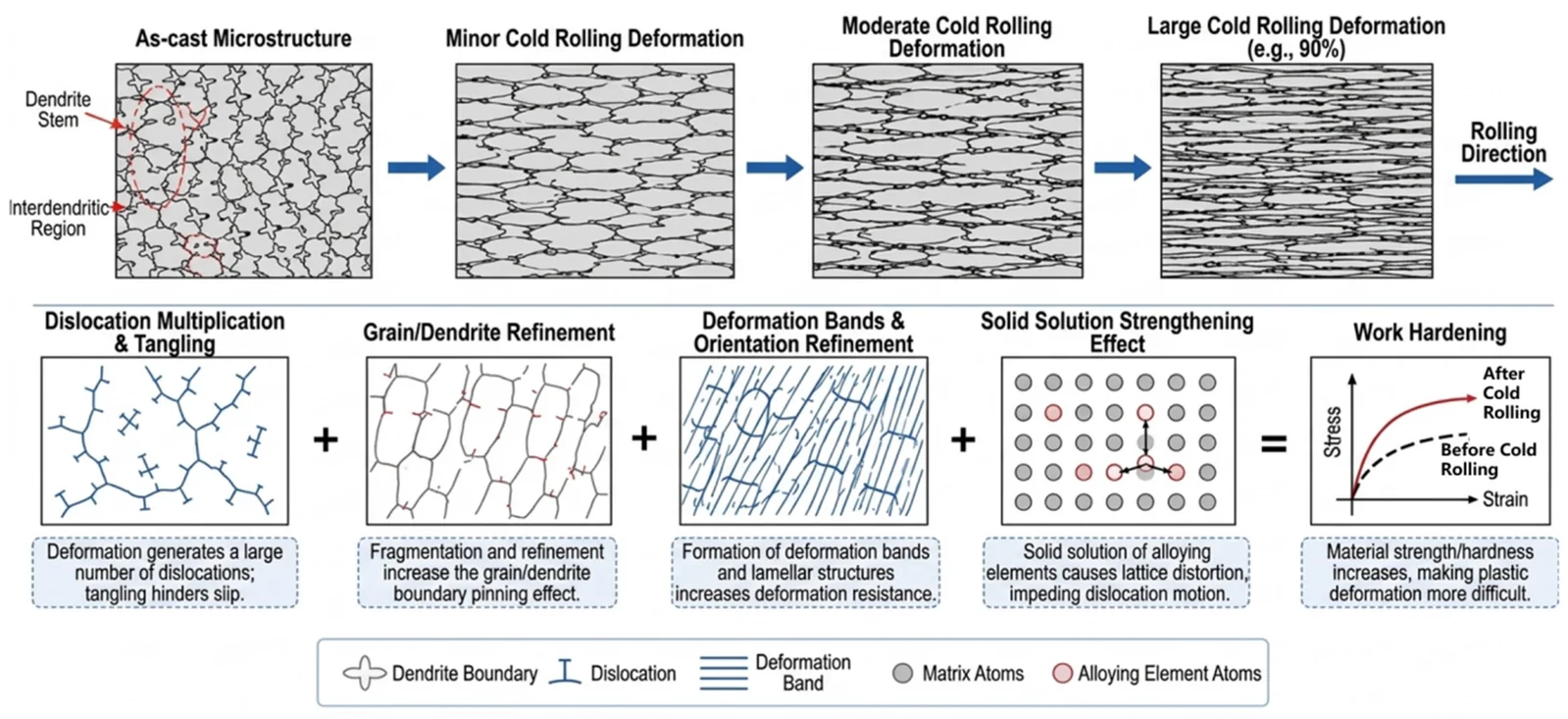
Schematic illustration of microstructural evolution and possible strengthening mechanisms during cold rolling.

As shown in Figure 10, the microstructural evolution of the initial state dendritic structure and the corresponding strengthening mechanisms during cold rolling can be systematically elucidated through multi-stage structural transformations. Only the initial and 90–reduced states were experimentally characterized, the minor deformation and moderate deformation stages are hypothesized based on the characteristics of cold rolling deformation [36,37]. In the initial state, the alloy consists of coarse primary dendrite trunks and interdendritic regions. As the degree of cold rolling progresses from minor and moderate deformation to a severe 90% reduction, the original equiaxed dendrites are progressively elongated, flattened, and severely fragmented along the rolling direction, ultimately converting into highly directional fibrous and lamellar structures. This microstructural transformation may be driven by the synergistic integration of four key mechanisms. Extensive dislocation multiplication and tangling that effectively impede further slip, dramatic microstructural refinement that significantly enhances the boundary pinning effect, the formation of high-density deformation bands that elevate localized deformation resistance, and pronounced solid solution strengthening caused by severe lattice distortion from alloying elements like Al. The combined action of these microstructural modifications and high-density defect accumulations fundamentally leads to a substantial enhancement in the work hardening capacity of the alloy, manifested macroscopically as a sharp increase in hardness.

## 4. Conclusions

The microstructural evolution and hardness variations of Fe_16_Co_28_Cr_40-x_Ni_16_Al_x_ (x = 8, 10) HEAs subjected to 90% cold rolling and 800 °C annealing were systematically investigated. The main conclusions drawn from this study are as follows:

(1) Al8 and Al10 initial state alloys exhibit typical FCC dendritic structures. The higher Al content in the Al10 alloy exacerbates elemental segregation during solidification and increases the initial hardness to 213.1 HV due to enhanced solid solution strengthening, compared to 192.9 HV in the Al8 alloy.

(2) After 90% cold rolling, the original dendrites of the Al8 alloy undergo thorough fragmentation and refinement, reducing the dendritic feature size to 2.0 μm and causing a drastic hardness increase to 351.6 HV. In contrast, the Al10 alloy exhibits higher matrix deformation resistance, retaining continuous, elongated dendritic bands with a dendritic feature size of 4.3 μm and reaching a hardness of 334.0 HV.

(3) Severe cold rolling fundamentally alters the subsequent annealing behavior at 800 °C. The structural evolution of the Al8 alloy follows a fragmentation, refinement, and dispersion path, whereas the Al10 alloy follows an elongation, banding, and localized refinement trajectory. Furthermore, under this annealing process, the elevated Al content in the Al10 alloy enhances matrix stability and effectively suppresses dendrite growth during annealing.

(4) During 800 °C annealing, the cold-rolled Al8 alloy initially experiences recovery softening, hardness dropping to 328.3 HV at 15 min due to stored energy release, followed by a slight rebound. Conversely, the cold-rolled Al10 alloy exhibits a continuous hardening trend, surging to 364.4 HV after 30 min. This indicates that, under this annealing process, the strong solid solution strengthening and superior microstructural stability in the Al10 alloy may override the softening effects brought by structural recovery.

## Figures and Tables

**Table 1:** Calculated results of Fe_16_Co_28_Cr_40-x_Ni_16_Al_x_ (x = 8, 10) HEA characteristic parameters.

Alloy	∆S_mix_ (K·mol)	∆H_mix_ (KJ/mol)	δ (%)	VEC
Al8	1.51R	−7.87	3.97	7.56
Al10	1.53R	−8.70	4.38	7.50

## Data Availability

The original contributions presented in this study are included in the article. Further inquiries can be directed to the corresponding authors.
